# Assay of anti-cancer drugs in tissue culture: relationship of relapse free interval (RFI) and in vitro chemosensitivity in patients with malignant cerebral glioma.

**DOI:** 10.1038/bjc.1985.75

**Published:** 1985-04

**Authors:** D. G. Thomas, J. L. Darling, E. A. Paul, T. J. Mott, J. N. Godlee, J. S. Tobias, L. G. Capra, C. D. Collins, C. Mooney, T. Bozek

## Abstract

One hundred and seventeen patients with cerebral glioma (Kernohan grades III and IV) were treated with adjuvant chemotherapy using procarbazine (PCB), CCNU and vincristine (VCR) following whole head irradiation. Cell cultures were prepared from 40 patients in this series and their sensitivity to each cytotoxic drug was assessed in a mictotitration assay with 35 S-methionine incorporation as the end point. Twenty-two of forty (55%) patients responded to PCB and/or CCNU in vitro, and sensitivity to these drugs was linked with increased RFI, whilst sensitivity to VCR was not. The RFI of patients who had responded to PCB or CCNU in vitro was significantly longer than the RFI of patients whose tumours failed to respond in vitro or patients who had not been tested. There was no difference in sex ratio, extent of operation, radiation dose and degree of steroid cover between responders, non-responders and untested groups. Grade III tumours tended to be more sensitive in vitro than grade IV tumours. The age of patients also influenced in vitro chemosensitivity. Patients with chemosensitive tumours in vitro tended to be younger than patients with insensitive tumours in vitro. Further statistical analysis, taking into account these prognostic factors, indicated an association between chemosensitivity in vitro and RFI.


Br. J. Cancer (1985), 51, 525–532

### Assay of anti-cancer drugs in tissue culture: Relationship of relapse free interval (RFI) and *in vitro* chemosensitivity in patients with malignant cerebral glioma

D.G.T. Thomas[1], J.L. Darling[1], E.A. Paul[2], T.J. Mott[3], J.N. Godlee[4], J.S. Tobias[4], L.G. Capra[5], C.D. Collins[6], C. Mooney[4], T. Bozek[4], G.P. Finn[4], S.O. Arigbabu[1]*, D.E. Bullard[1]†, N. Shannon[1] & R.I. Freshney[7]

[1]*Gough-Cooper Department of Neurological Surgery, Institute of Neurology, Queen Square, London;* [2]*Computing and Statistics Unit, Institute of Neurology, Queen Square, London;* [3]*The Ipswich Hospital, Ipswich;* [4]*University College Hospital, London;* [5]*St Luke's Hospital, Guildford;* [6]*St Thomas's Hospital, London; and* [7]*Department of Clinical Oncology, University of Glasgow, Glasgow, UK.*

**Summary** One hundred and seventeen patients with cerebral glioma (Kernohan grades III and IV) were treated with adjuvant chemotherapy using procarbazine (PCB), CCNU and vincristine (VCR) following whole head irradiation. Cell cultures were prepared from 40 patients in this series and their sensitivity to each cytotoxic drug was assessed in a mictotitration assay with 35 S-methionine incorporation as the end point. Twenty-two of forty (55%) patients responded to PCB and/or CCNU *in vitro,* and sensitivity to these drugs was linked with increased RFI, whilst sensitivity to VCR was not. The RFI of patients who had responded to PCB or CCNU *in vitro* was significantly longer than the RFI of patients whose tumours failed to respond *in vitro* or patients who had not been tested. There was no difference in sex ratio, extent of operation, radiation dose and degree of steroid cover between responders, non-responders and untested groups. Grade III tumours tended to be more sensitive *in vitro* than grade IV tumours. The age of patients also influenced *in vitro* chemosensitivity. Patients with chemosensitive tumours *in vitro* tended to be younger than patients with insensitive tumours *in vitro.* Further statistical analysis, taking into account these prognostic factors, indicated an association between chemosensitivity *in vitro* and RFI.

Malignant cerebral gliomas represent a formidable clinical challenge despite over 50 years of intensive clinical and experimental investigation. While radiotherapy and chemotherapy both prolong useful life (Walker & Gehan, 1976; Bloom, 1982) the long-term prognosis for patients with this disease remains consistently poor.

There is widespread morphological variation in gliomas of similar clinical malignancy (Russell & Rubinstein, 1977) and recent evidence suggests that this variability may extend to characteristics other than morphology (Shapiro *et al.,* 1981; Bigner *et al.,* 1981; Bradley *et al.,* 1978; Bullard *et al.,* 1981*a*). It is possible that sensitivity to cytotoxic drugs varies between individual tumours (Barranco *et al.,* 1973; Kimball & Brattain, 1980) and that this is an explanation for the clinical observation of the wide variation in response to chemotherapy amongst patients with otherwise similar tumours. Attempts to investigate this problem in the laboratory using a variety of *in vitro* and *in vivo* techniques (Gazso & Afra, 1969; Mealey *et al.,* 1974; Kornblith & Szypko, 1978; Easty & Wylie, 1963; Saez *et al.,* 1977; Rosenblum *et al.,* 1978, 1983; Shapiro *et al.,* 1979; Bullard *et al.,* 1981*b*) have all demonstrated that human gliomas display considerable variation in chemosensitivity. In this report we present data produced with a rapid 35 S-methionine uptake assay (Darling & Thomas, 1983; Freshney & Dendy, 1983) where the end-point has been measured by scintillation autofluorography (Thomas *et al.,* 1979; Morgan *et al.,* 1983) which permits a rapid, objective comparison of *in vitro* and clinical data.

### Materials and methods

### *Cell culture and chemosensitivity assay*

Biopsy samples were taken at operation for routine diagnostic neuropathological examination. Histology was reported by consultant neuropathologists at The National Hospitals, Queen Square or Maida Vale as either grade III or grade IV malignant glioma. Of 48 biopsies submitted for tissue culture,

Correspondence: D.G.T. Thomas.
*Present address: Neurosurgical Unit, Department of Surgery, Lagos University Teaching Hospital, Lagos, Nigeria.
†Present address: Department of Neurosurgery, Division of Surgery, Duke University Medical Center, Durham, North Carolina, USA.
Received 5 June 1984; and in revised form 10 December 1984.

© The Macmillan Press Ltd., 1985

40 (83%) were evaluable for chemosensitivity. Two (4%) became contaminated and 6 (12.5%) cases failed to grow in culture. Samples were collected in Ham's F-10 culture medium supplemented with $100\,u\,ml^{-1}$ penicillin, $100\,\mu g\,ml^{-1}$ streptomycin and buffered with 20 mM HEPES. Cell culture and the chemosensitivity assay have both been described in detail previously (Thomas *et al.*, 1979; Morgan *et al.*, 1983). Briefly, cells at passage level 1 or 2 were diluted to $1\text{–}5 \times 10^5$ cells $ml^{-1}$ inoculated onto 96-well microtitration plates ($1\text{–}5 \times 10^4$ cells/well) and incubated at 37°C for 24–72 h. Stock drug solutions were made up as follows: VCR (Oncovin, Eli Lilly) $100\,\mu g\,ml^{-1}$; PCB (Natulan, Roche) $500\,\mu g\,ml^{-1}$ in Ham's F-10 medium and CCNU (Lundbeck) $100\,\mu g\,ml^{-1}$ in absolute ethyl alcohol, and subsequently stored at −80°C. Drug solutions were all diluted in Ham's F-10 plus 10% foetal calf serum, with $50\,u\,ml^{-1}$ penicillin, $50\,\mu g\,ml^{-1}$ streptomycin and buffered with 20 mM HEPES. At the time of testing, 0.2 ml of each drug dilution was added to the appropriate wells and renewed 24 and 48 h later to give a total exposure time of 72 h. The concentration ranges of drugs used in this study has been published together with their relationship to the *in vivo* concentrations attainable (Morgan *et al.*, 1983). Finally, the drugs were removed and each well gently washed and filled with 0.2 ml of fresh growth medium. After 3–5 days recovery period, 0.1 ml of $2\,\mu Ci\,ml^{-1}$ of 35 S-methionine was added to each well, and incubated for 4–18 h. The plates were then washed in Hanks' buffered saline, fixed in methanol, extracted in trichloroacetic acid, washed in tap water and dried in methanol. Toluene-based scintillation fluid (50 $\mu$l) was added to each well and the plates were dried by centrifugation. A sheet of X-ray film was then placed under each plate and exposed for between 24 h and 2 weeks. Each column of the developed negative was scanned with a densitometer and the $ID_{50}$ (the dose of the drug which inhibited protein synthesis by 50%) was determined from the densitometer traces (Thomas *et al.*, 1979).

Previous studies with HeLa cells (Freshney *et al.*, 1975) have shown that prolonged exposure to drugs followed by recovery were necessary to obtain stable $ID_{50}$ values for most drugs, especially phase specific agents. Therefore, in order to minimise internal variation in the assay, cultures were sampled after 3–5 days recovery but before density limitation of growth occurred (Morgan *et al.*, 1983). At this point, the $ID_{50}$ measured by clonogenicity and microtitration assay correlate well (Morgan *et al.*, 1983). The $ID_{50}$ of each glioma cell culture was determined and compared to a panel of 123 cultures derived from tumours of the same histology that had been assayed for chemosensitivity to PCB, CCNU and VCR in this laboratory using the same assay. Sensitivity was defined as an $ID_{50}$ below the median $ID_{50}$ of the panel of cultures. If the $ID_{50}$ was above the median for the group then the culture was designated a non-responder.

*Patients*

One hundred and seventeen patients (see Table II) with histologically verified malignant gliomas were treated post operatively with a combination of radiotherapy and chemotherapy. Radiation was administered either to the whole brain, or to a complete hemisphere with appropriate overlap to include possible extension into the opposite hemisphere. The tumour minimum dose was 40–60 Gy. Steroid therapy with dexamethasone or betamethasone was used as necessary to control raised intracranial pressure, but at the lowest maintenance dose consistent with good medical management and stopped, in most cases, as soon as radiation treatment had been completed. It was only reintroduced when signs and symptoms were indicative of tumour recurrence. From 2–6 weeks after the completion of radiation therapy, patients began chemotherapy, on an outpatient basis, consisting of VCR, $1.4\,mg\,m^{-2}$ as a single dose, CCNU, $80\,mg\,m^{-2}$ as a single dose, orally, and PCB, orally, $100\,mg\,m^{-2}$ per day over 10 days. Patients were reviewed clinically and haematologically every 6 weeks on an outpatient basis. Patients were treated for 12 cycles over an 18-month period. Computerized tomography (CT) was performed routinely approximately every 3 months during active therapy and every 6 months following its completion. The time from surgery until disease progression was termed the relapse free interval (RFI). Disease progression was defined as a marked deterioration in clinical status, which was in most, but not all cases, accompanied by a worsening in the CT scan. The criteria for deterioration used in this study were similar to those described by Levin *et al.* (1977) except that electroencephalographic recordings and radionuclide scanning were not routinely performed. Relapse free interval was chosen in this study rather than survival as extensive use of high dose glucocorticoids to control cerebral oedema can prolong survival considerably even in the face of obvious tumour recurrence. To be included in this study, all patients must have received a full course of radiotherapy and at least one course of chemotherapy. Patients were selected for the study purely on clinical grounds and it was not necessary for a tissue culture sample to have been taken or a chemosensitivity assay to have been carried out for a patient to receive chemotherapy. All clinical/*in vitro* correlations were carried out retrospectively, and no attempt was made to influence chemotherapy using the results of the assay.

### Results

Each patient whose cells were assayed was designated a responder or non-responder to a particular drug on the criteria described in the methods section. 14/40 (35%) of patients responded *in vitro* to CCNU, 17/40 (42%) of patients responded to PCB and 16/40 (40%) of patients responded to VCR. The length of relapse free interval was compared for those patients who were responders to a particular drug and those who were not. The comparison of relapse free intervals were based on the Mantel–Cox test (Mantel, 1966) for the comparison of censored survival times and were computed using Biomedical Data Program (BMDP) 1L. Table I shows the median relapse free intervals and the quartiles of patients who had sensitive or insensitive cultures *in vitro* for each of the 3 drugs (linear smoothing was used to estimate the percentiles, Miller, 1981). The differences in RFIs between the PCB and CCNU responders and non-responders *in vitro* were significant ($P=0.02$ and $P=0.01$ respectively). The differences between the RFIs in the case of responders or non-responders to vincristine were not significantly different ($P=0.5$). A Cox regression model was fitted (using BMDP 2L) to look at the combined effects of sensitivity to each drug on RFI. This confirmed that only sensitivity to PCB and CCNU was associated with increased RFI.

*In vitro* chemosensitivity to either PCB or to CCNU was related to RFI while *in vitro* sensitivity to VCR was not. Glioma patients who had undergone chemotherapy were divided into 3 groups. The first group consisted of 22 responders *in vitro* to either PCB or CCNU or to both (Group A). The second group consisted of 18 patients who did not respond to either of these drugs *in vitro* (Group B). The third group comprised 77 patients who had not been tested for chemosensitivity (Group C). Figure 1 shows the Kaplan–Meier survival plots (Kaplan & Meier, 1958) for the relapse-free intervals of these groups. The chemosensitive group (A) remained relapse-free for longer than the non-chemosensitive group B (Mantel–Cox test, $P<0.0001$). The RFIs of those patients whose cells had been tested *in vitro* (A+B) were compared with the RFIs for the untested group, C, and no significant difference was found ($P=0.28$).

To ascertain whether the improved RFI in the chemosensitive group could be explained as a function of variation in other prognostic factors, a comparison was made between the groups on a number of possible prognostic factors (Table II). Sex, type or extent of operation, amount of radiation, steroids and tumour site were not significantly different in the 3 groups. There were however differences in age and grade. The untested group (C) were older than the tested group (A+B) and patients who responded *in vitro* to either CCNU or PCB (A) tended to be younger than those patients who did not respond (B), although this difference did not reach statistical significance. Although there was no difference in the proportion of grade III and grade IV tumours between the tested groups (A+B) and the untested groups (C), there were fewer grade III tumours in the non-chemosensitive group (B) than in the chemosensitive group (A): 16/22 (73%) sensitive tumours were grade III, but only 6/22 (27%) sensitive tumours were grade IV. Conversely, only 4/18 (22%) resistant tumours were grade III whilst 14/18 (78%) of these tumours were grade IV.

Further tests were done to see, in the whole sample, the effect of each of the prognostic factors on RFI (Table III). Age, grade and site of tumour were each associated with RFI. Patients with parietal tumours had shorter RFIs. However, within group C (the untested patients) there was no significant difference in RFI between patients with tumours at different sites. The proportion of patients with parietal tumours was similar in group

**Table I** Median relapse free intervals of patients with sensitive and insensitive cell cultures

| | | *Median relapse free intervals (d)* | | |
|---|---|---|---|---|
| | *Quartiles* | *CCNU* | *VCR* | *PCB* |
| Non-chemosensitive *in vitro* | 75 | 178 | 185 | 164 |
| | median | 253 | 331 | 233 |
| | 25 | 537 | 527 | 374 |
| Chemosensitive *in vitro* | 75 | 332 | 212 | 387 |
| | median | 857 | 539 | 609 |
| | 25 | 1438 | 1120 | 837 |

**Figure 1** Kaplan–Meier survival plots of the relapse free intervals of patients with malignant gliomas who had been treated with the PCV protocol and were sensitive to PCB and/or CCNU *in vitro* (Group A), those who were insensitive to either of these drugs (Group B) and those patients who had not been tested *in vitro* (Group C).

**Table II** Characteristics of patients in the chemosensitive, not chemosensitive and not tested groups

| | | *Tested for chemosensitivity* | | *Untested for chemosensitivity* | *Comparison of tested* vs *not tested groups* | *Comparison of chemosensitive* vs *not chemosensitive* |
|---|---|---|---|---|---|---|
| | | *Sensitive* (*group A*) | *Insensitive* (*group B*) | (*Group C*) | (*Group A* + *B* vs *group C*) | (*Group A* vs *group B*) |
| | | | | | (*t*-test) | |
| Sample size | | 22 | 18 | 77 | *P*= | *P*= |
| Age at diagnosis mean | | 42.9 | 49.4 | 51.7 | 0.019 | 0.1408 |
| s.d. | | 12.1 | 15.2 | 12.0 | | |
| | | Percentage of groups | | | ($\chi^2$-test) | |
| Sex | Male | 55 | 78 | 61 | 0.82 | 0.23 |
| | Female | 45 | 22 | 39 | | |
| Histology | Grade III | 73 | 22 | 40 | 0.42 | 0.0042 |
| | Grade IV | 27 | 78 | 60 | | |
| Tumour site | Frontal | 50 | 25 | 39 | 0.78 | 0.07 |
| | Temporal | 35 | 25 | 25 | | |
| | Parietal | 15 | 50 | 36 | | |
| Operation | Partial | 68 | 56 | 62 | 0.98 | 0.62 |
| | Lobectomy | 32 | 44 | 38 | | |
| Radiation | 0–4999 cGy | 74 | 67 | 76 | 0.76 | 0.98 |
| | 5000 + cGy | 26 | 33 | 24 | | |
| Steroids | Preoperative | 59 | 47 | 53 | 0.91 | 0.67 |
| | Pre & post op. | 41 | 53 | 47 | | |

**Table III** Variables examined for possible effect on relapse free interval (individual Mantel–Cox tests)

| | | *Total sample Median RFI (days)* | | *Untested group Median RFI (days)* | |
|---|---|---|---|---|---|
| | | | $P=$ | | $P=$ |
| Age | 0–30 | 746 | | 504 | |
| | 31–50 | 493 | 0.0001 | 532 | 0.0007 |
| | 51+ | 223 | | 210 | |
| Sex | Male | 287 | 0.84 | 259 | 0.95 |
| | Female | 350 | | 337 | |
| Histology | Grade III | 397 | 0.001 | 350 | 0.05 |
| | Grade IV | 268 | | 259 | |
| Site | Frontal | 385 | | 350 | |
| | Temporal | 421 | 0.05 | 371 | 0.41 |
| | Parietal | 231 | | 233 | |
| Operation | Partial | 277 | 0.98 | 233 | 0.40 |
| | Lobectomy | 384 | | 421 | |
| Radiation | 0–4999 cGy | 340 | 0.34 | 301 | 0.61 |
| | 5000+cGy | 493 | | 337 | |
| Steroids | Preoperative | 385 | 0.16 | 350 | 0.34 |
| | Pre & post op. | 233 | | 222 | |

C and those patients who had been tested (groups A and B). However, amongst those tested a higher proportion of parietal tumours were not sensitive *in vitro*.

For groups A and B, Cox's proportional hazard model (PHM) (Cox, 1972) was fitted using a forward stepwise procedure and including in the model, age, grade, site of tumour, chemosensitivity and including all interactions up to the third order. The final model showed that when all other factors were taken into account chemosensitivity was still related to longer RFI.

To check that the similarity in RFI in the tested and untested groups was not because an actual difference was masked by variations in age, grade or site, a Cox PHM was fitted including these factors. When the effect of age, grade and site were taken into account there was still no difference in RFI between the tested (A+B) and the untested (C) groups.

### Discussion

The triple agent regimen of PCB, CCNU and VCR has evolved from initial studies by Gutin *et al.* (1975) and Shapiro & Young (1976) who combined three agents which had been reported as modestly successful single agents for the treatment of glioma. All three agents are capable of passing the blood-brain barrier and therefore might be expected to pass not only into the body of the tumour but also into the tumour periphery with its infiltrating edge. Experimental evidence (Rosenblum *et al.,* 1976) suggests that administering cycle specific agents such as vincristine after treatment with CCNU and PCB might enhance maximum cell kill during a period when tumour cells should be rapidly proliferating.

From our study we have demonstrated that patients do not respond to these agents in the same way. It is possible to divide patients into two groups, those whose tumours respond to PCB or CCNU *in vitro* and those whose tumours do not. Morphologically similar tumours do not always uniformly respond to single therapeutic agents. Usually this is ascribed to vague factors such as host-tumour interaction or differences in regional drug delivery. Recent experimental work, has, however, strongly suggested that the intrinsic variation in chemosensitivity among individual tumours may be one factor which is responsible for these patterns of response. Detailed *in vitro* studies (Darling & Thomas, in preparation) indicate that glioma cell cultures demonstrate considerable heterogeneity in response to not only PCB, CCNU and VCR but also to other agents such as adriamycin, VP 16-213, AZQ, bleomycin and 5-FU. Shapiro *et al.* (1981) and Yung *et al.* (1982) have demonstrated that clones from a single glioma biopsy display differing chemosensitivities *in vitro*.

Kornblith and colleagues (1978, 1981) have demonstrated considerable variation in the sensitivity of glioma cultures to BCNU. In their study 5/14 patients were insensitive to BCNU and none of these patients responded clinically to the drug. The remaining 9 patients did respond *in vitro*, but only 6 of them demonstrated clinical response. Using a cloning assay, Rosenblum and colleagues (1981, 1983) have found similar variation in *in vitro* chemosensitivity to BCNU. From a group of 15 patients, 8 were insensitive to BCNU *in vitro* and 7 were sensitive. As in Kornblith's study all patients who failed to respond *in vitro* also failed to respond clinically, and only 3 of the sensitive patients responded to BCNU clinically. In our study 22/40 (55%) of patients were sensitive to either PCB or CCNU and 18/40 patients failed to respond to these drugs. It is also apparent that patients who had tumours sensitive to PCB and/or CCNU *in vitro* had significantly longer RFIs than those patients who did not respond to either of these drugs, or who were not tested *in vitro*. Response to VCR *in vitro* did not influence RFI.

The apparent ineffectiveness of vincristine in prolonging relapse free interval is rather unexpected in light of a number of reports indicating its modest effect as a single agent (Edwards *et al.*, 1980). Vincristine does appear to be particularly effective against rapidly growing intracranial tumours such as medulloblastomas (Crafts *et al.*, 1978). In malignant gliomas which have relatively small growth fractions (often $<10\%$, Steel, 1980) vincristine may well be less effective than cycle non-specific drugs like nitrosoureas and procarbazine. As the chemosensitivity assay uses exponentially dividing cells *in situ* sensitivity may be over-predicted because of the relative insensitivity on non-cycling cells *in situ*. As CCNU and procarbazine are not restricted in their effectiveness by cell cycle constraints the *in situ* effectiveness of these drugs will be demonstrable in either cycling or non-cycling cells.

It may be that vincristine cannot penetrate into the actively growing periphery of the tumour because of a partially intact blood–brain barrier. Evidence from animal experiments using intra-cranial tumour models indicates that the brain adjacent to the tumour (BAT) is less permeable to hydrophilic molecules than normal brain while remaining freely permeable to lipophilic drugs (Levin *et al.*, 1975). This might indicate that vincristine cannot attain sufficiently high concentrations *in situ* to be effective.

It is unlikely that the reason for the apparent ineffectiveness of vincristine is due to a rapid overgrowth of resistant cells or a rapid increase in *de novo* resistant cells as our experiments with glioma cell cultures indicate that cells treated with vincristine remain sensitive to this drug throughout a recovery period in fresh growth medium while cultures recover rapidly from treatment with procarbazine and CCNU.

Histological grade did seem to effect *in vitro* chemosensitivity. Cultures prepared from grade III tumours were more likely to respond to PCB or CCNU than cultures prepared from grade IV tumours. Although grade III tumours are known to be less aggressive in their clinical history than the more malignant grade IV tumours, less is known about therapeutic differences between these groups of tumours. Bloom (1982) has reported that clinically grade III astrocytomas tend to be more sensitive to treatment with nitrosoureas than grade IV astrocytomas and Levin *et al.* (1980) has shown that glioblastoma multiforme is less sensitive to treatment with PCB, CCNU and VCR than gliomas of lower grades of malignancy. *In vitro*, there is some evidence that cultures from grade III tumours are more sensitive to BCNU than cultures derived from grade IV tumours (Kornblith *et al.*, 1981). The effect of patient age on *in vitro* chemosensitivity has already been examined by Rosenblum *et al.* (1982) who found an inverse correlation between sensitivity to BCNU *in vitro* and the patients age. Patients with sensitive cells tended to be younger than those patients with resistant cells. From our data it is also apparent that within the tested groups (A + B), fewer parietal tumours are sensitive *in vitro* (Table II), and this results in an overall poorer prognosis for patients with parietal tumours in the group as a whole. A detailed examination of the Brain Tumor Study Group data has failed to confirm that patients with parietal tumours have a worse prognosis than patients with tumours in other locations (Byar *et al.*, 1983).

The association between *in vitro* chemosensitivity and RFI demonstrated in this study is in accordance with studies which have shown a correlation between *in vitro* drug sensitivity and survival of patients with ovarian cancer (Alberts, 1981) and multiple myeloma (Durie *et al.*, 1983). In a study of patients with relapsed ovarian cancer, Alberts (1981) has shown that those patients treated with drugs to which they were sensitive in the assay had significantly longer survival times than those patients who were either resistant in the assay or sensitive but were treated empirically with drugs not recommended by the assay. In this study, care was taken to see that these differences in survival were not simply due to an imbalance of prognostic factors between groups.

The present study found that length of RFI of patients with malignant gliomas undergoing adjuvant chemotherapy is linked with sensitivity to PCB and CCNU *in vitro*. Although other prognostic signs do influence clinical outcome, statistical analysis confirms *in vitro* chemosensitivity as a factor in determining relapse free interval. The next stage is to use this assay to carry out a prospective trial of chemosensitivity testing for malignant gliomas.

The authors are grateful to the physicians and other surgeons who referred patients and to Dr R. Souhami and Dr C. Hawkes for helpful discussions. The authors would like to thank Mr C.P. Chalmers, Department of Statistics, Birbeck College, London for helpful comments on the statistical aspects of this work. The technical assistance of B.A. Watkins and Maria Hine is gratefully acknowledged. This work is supported by grants from the Cancer Research Campaign and the Brain Research Trust to DGTT and JLD.

### References

ALBERTS, D.S. (1981). Improved survival for relapsing ovarian cancer (OVCA) patients (PTS) using the human tumor stem cell assay (HTSCA) to select chemotherapy (CRX). *Stem Cells*, **1**, 294.

BARRANCO, S.C., DREWINKO, B. & HUMPHREY, R.M. (1973). Differential response by human melanoma cells to 1,3,-bis-(2-chloroethyl)-1-nitrosourea and bleomycin. *Mutat. Res.*, **19**, 277.

BIGNER, S.H., BULLARD, D.E., PEGRAM, C.E., WIKSTRAND, C.W. & BIGNER, D.D. (1981). Relationship of *in vitro* morphologic and growth characteristics of established human glioma-derived cell lines to their tumorigenicity in athymic mice. *J. Neuropathol. Exp. Neurol.*, **40**, 390.

BLOOM, H. J. G. (1982). Intracranial tumors: Response and resistance to therapeutic endeavours, 1970–1980. *Int. J. Radiat. Oncol. Biol. Phys.*, **8**, 1083.

BRADLEY, N.J., BLOOM, H.J.G., DAVIES, A.J.S. & SWIFT, S.M. (1978). Growth of human gliomas in immune-deficient mice: A possible model for preclinical therapy studies. *Br. J. Cancer*, **38**, 263.

BULLARD, D.E., BIGNER, S.H. & BIGNER, D.D. (1981*a*). The morphologic response of cell lines derived from human gliomas to dibutryl adenosine 3′:5′-cyclic monophosphate. *J. Neuropathol. Exp. Neurol.*, **40**, 230.

BULLARD, D.E., SCHOLD, S.C., BIGNER, S.H. & BIGNER, D.D. (1981*b*). Growth and chemotherapeutic response in athymic mice of tumours arising from human glioma-derived cell lines. *J. Neuropathol. Exp. Neurol.*, **40**, 410.

BYAR, D.P., GREEN, S.B. & STRIKE, T.A. (1983). Prognostic factors for malignant gliomas. In: *Oncology of the Nervous System*. (Ed. Walker), Boston: Martinius Nijhoff, p. 379.

COX, D.R. (1972). Regression models and life tables. *J. R. Stat. Soc., Series B*, **34**, 187.

CRAFTS, D.C., LEVIN, V.A. EDWARDS, M.S. PISCHER, T.L. & WILSON, C.B. (1978). Chemotherapy of recurrent of medulloblastoma with combined procarbozine, CCNU and vincristine. *J. Neurosurg.*, **49**, 589.

DARLING, J.L. & THOMAS, D.G.T. (1983). Results obtained using assays of intermediate duration and clinical correlations. In: *Human Tumour Drug Sensitivity Testing In Vitro – Techniques and Clinical Applications*. (Eds. Dendy & Hill), London: Academic Press, p. 269.

DURIE, B.G.M., YOUNG, L.A. & SALMON, S.E. (1983). Human myeloma *in vitro* colony growth: Interrelationships between drug sensitivity, cell kinetics and patient survival data. *Blood*, **61**, 929.

EASTY, D.M. & WYLIE, J.A.H. (1963). Screening of 12 gliomata against chemotherapeutic agents *in vitro*. *Br. Med. J.*, **i**, 1589.

EDWARDS, M.S., LEVIN, V.A. & WILSON, C.B. (1980). Brain tumour chemotherapy: An evaluation of agents in current use for phase I and II trials. *Cancer Treat. Rep.*, **64**, 1179.

FRESHNEY, R.I. & DENDY, P.P. (1983). Culture methods for assays of intermediate duration. In: *Human Tumour Drug Sensitivity Testing In Vitro – Techniques and Clinical Applications*. (Eds. Dendy & Hill), London: Academic Press, p. 69.

FRESHNEY, R.I., PAUL, J. & KANE, I.M. (1975). Assay of anti-cancer drugs in tissue culture: Conditions affecting their ability to incorporate $^3$H-leucine after drug treatment. *Br. J. Cancer*, **31**, 89.

GAZSO, L.R. & AFRA, D. (1969). Study on the effect of actinomycins in tissue cultures from human brain tumours. *Acta Neurochir (Wein)*, **21**, 139.

GUTIN, P.H. & 6 others. (1975). Phase II study of procarbazine, CCNU, and vincristine combination chemotherapy in the treatment of malignant brain tumors. *Cancer*, **35**, 1389.

KAPLAN, E.L. & MEIER, P. (1958). Nonparametric estimation from incomplete observations. *J. Am. Stat. Assoc.*, **53**, 457.

KIMBALL, P.M. & BRATTAIN, M.G. (1980). Isolation of a cellular subpopulation from a human colonic carcinoma cell line. *Cancer Res.*, **40**, 1574.

KORNBLITH, P.L., SMITH, B.H. & LEONARD, L.A. (1981). Response of cultured human brain tumors to nitrosoureas: Correlation with clinical data. *Cancer*, **47**, 255.

KORNBLITH, P.L. & SZYPKO, P.E. (1978). Variations in response of human brain tumors to BCNU *in vitro*. *J. Neurosurg.*, **48**, 580.

LEVIN, V.A., FREEMAN-DOVE, M. & LANDHAL, H.D. (1975). Permeability characteristics of brain adjacent to tumours in rats. *Arch. Neurol.*, **35**, 758.

LEVIN, V.A., CRAFTS, D.C., NORMAN, D.N., HOFFER, P.B., SPIRE, J.P. & WILSON, C.B. (1977). Criteria for evaluating patients undergoing chemotherapy for brain tumours. *J. Neurosurg.*, **47**, 329.

LEVIN, V.A., EDWARDS, M.S., WRIGHT, D.C. & 4 others. (1980). Modified procarbazine, CCNU and vincristine (PCV 3) combination chemotherapy in the treatment of malignant brain tumors. *Cancer Treat. Rep.*, **64**, 237.

MANTEL, N. (1966). Evaluation of survival data and two new rank order statistics arising from its consideration. *Cancer Chemo. Rep.*, **50,** 163.
MEALEY, J., CHEN, T.T. & SHUPE, R. (1974). Response of cultured glioblastomas to radiation and BCNU chemotherapy. *J. Neurosurg.*, **41,** 339.
MILLER, R.G. (1981). *Survival Analysis*. Chichester: Wiley, p. 75.
MORGAN, D., FRESHNEY, R.I., DARLING, J.L., THOMAS, D.G.T. & CELIK, F. (1983). Assay of anticancer drugs in tissue culture: Cell cultures of biopsies from human astrocytoma. *Br. J. Cancer,* **47,** 205.
ROSENBLUM, M.L., GEROSA, M., DOUGHERTY, D. & 5 others (1982). Age related chemosensitivity of stem cells from human malignant brain tumours. *Lancet,* **i,** 885.
ROSENBLUM, M.L., GEROSA, M.A., WILSON, C.B. & 4 others. (1983). Stem cell studies of human malignant brain tumors: Part 1. Development of the stem cell assay and its potential. *J. Neurosurg.*, **58,** 170.
ROSENBLUM, M.L., KNEBEL, K.D., VASQUEZ, D.A. & WILSON, C.B. (1976). *In vivo* clonogenic tumor cell kinetics following 1,3-bis-(2-chloroethyl)-1-nitrosourea brain tumor therapy. *Cancer Res.*, **36,** 3718.
ROSENBLUM, M.L., VASQUEZ, D.A., HOSHINO, T. & WILSON, C.B. (1978). Development of a clonogenic cell assay for human brain tumors. *Cancer,* **41,** 2305.
RUSSELL, D.S. & RUBINSTEIN, L.J. (1977). *Pathology of Tumours of the Nervous System*. 4th Edition, London: Edward Arnold.
SAEZ, R.J., CAMPBELL, R.J. & LAWS, E.R. (1977). Chemotherapeutic trials on human malignant astrocytomas in organ culture. *J. Neurosurg.*, **46,** 320.
SHAPIRO, J.R., YUNG, W.K.A. & SHAPIRO, W.R. (1981). Isolation, karyotype and clonal growth of hetereogeneous subpopulations of human malignant gliomas. *Cancer Res.*, **41,** 2349.
SHAPIRO, W.R., BASLER, G.A., CHERNIK, N.L. & POSNER, J.B. (1979). Human brain tumor transplantation into nude mice. *J. Natl Cancer Inst.*, **62,** 447.
SHAPIRO, W.R. & YOUNG, D.F. (1976). Chemotherapy of malignant glioma with CCNU alone and CCNU combined with vincristine sulfate and procarbazine hydrochloride. *Trans. Am. Neurol. Assoc.*, **101,** 217.
STEEL, G.G. (1980). Growth kinetics of brain tumours. In: *Brain Tumours Scientific Basis, Clinical Investigation and Current Therapy,* (Eds. Thomas & Graham), London: Butterworths, p. 10.
THOMAS, D.G.T., DARLING, J.L., FRESHNEY, R.I. & MORGAN, D. (1979). *In vitro* chemosensitivity assay of human glioma by scintillation autofluorography. In: *Multidisciplinary Aspects of Brain Tumor Therapy*. (Eds. Paolette *et al.*), Elsevier/North-Holland Biomedical Press, p. 19.
WALKER, M.D. & GEHAN, E.A. (1976). Clinical studies in malignant gliomas and their treatment with the nitrosoureas. *Cancer Treat. Rep.*, **60,** 713.
YUNG, W.K.A., SHAPIRO, J.R. & SHAPIRO, W.R. (1982). Heterogeneous chemosensitivities of subpopulations of human glioma cells in culture. *Cancer Res.*, **42,** 992.


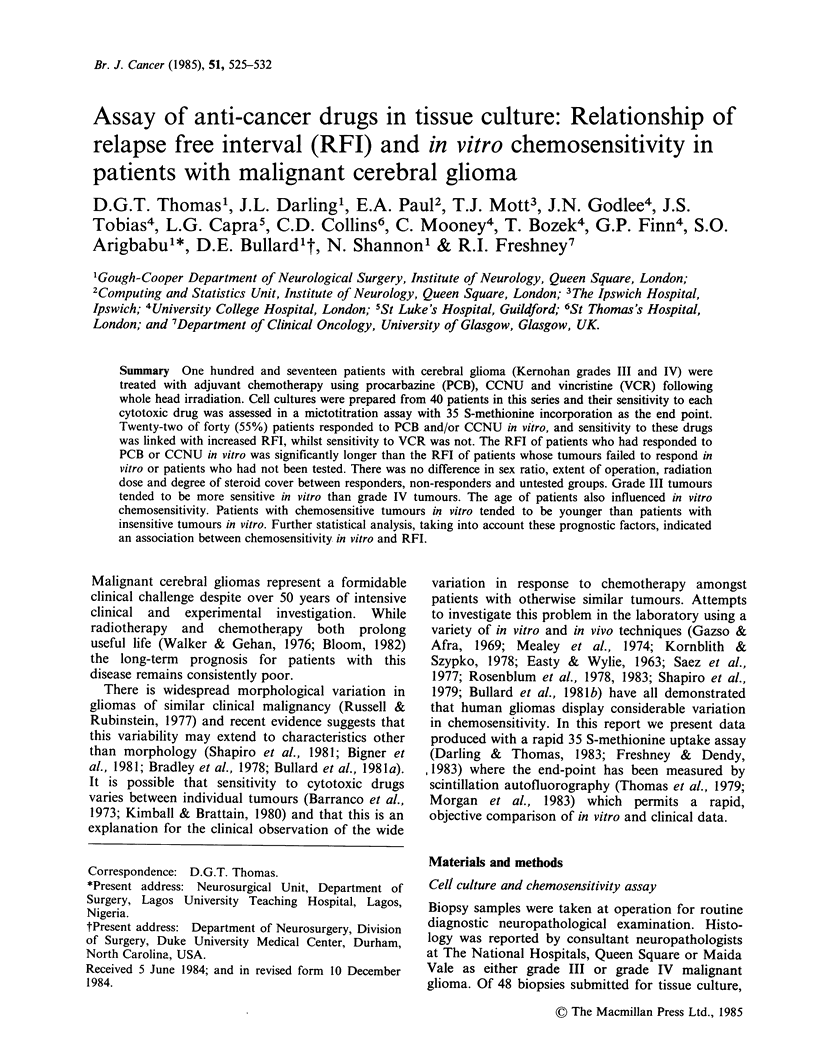

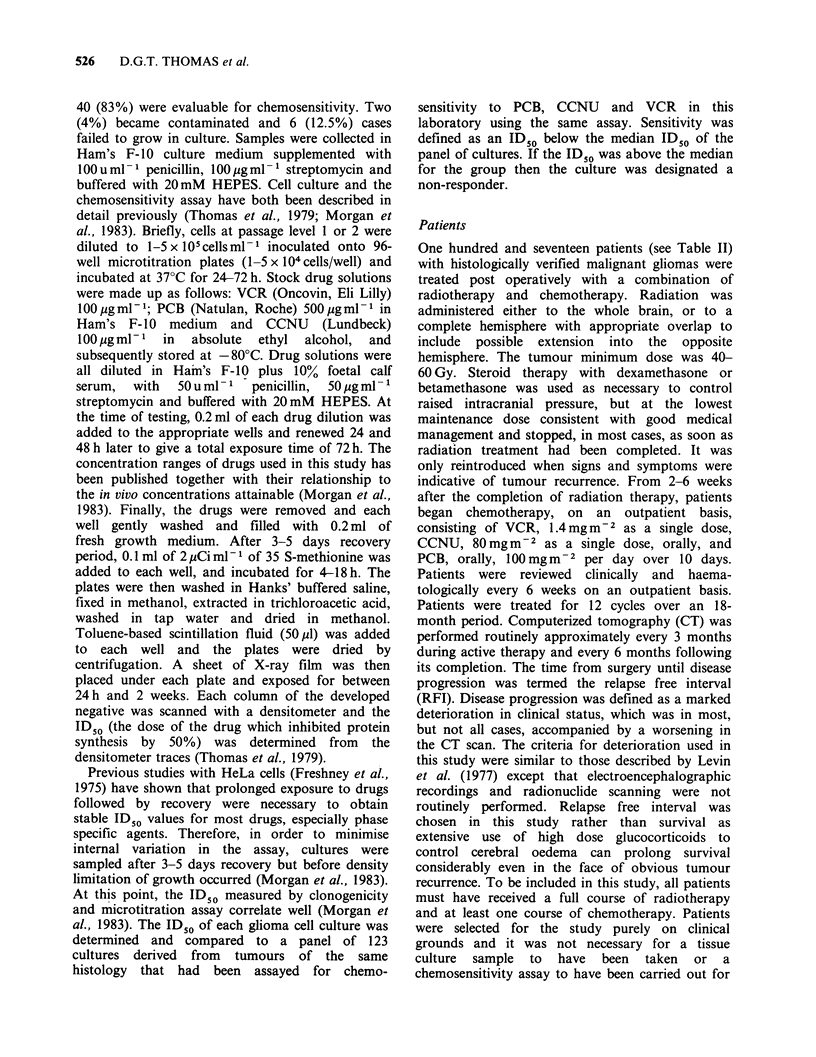

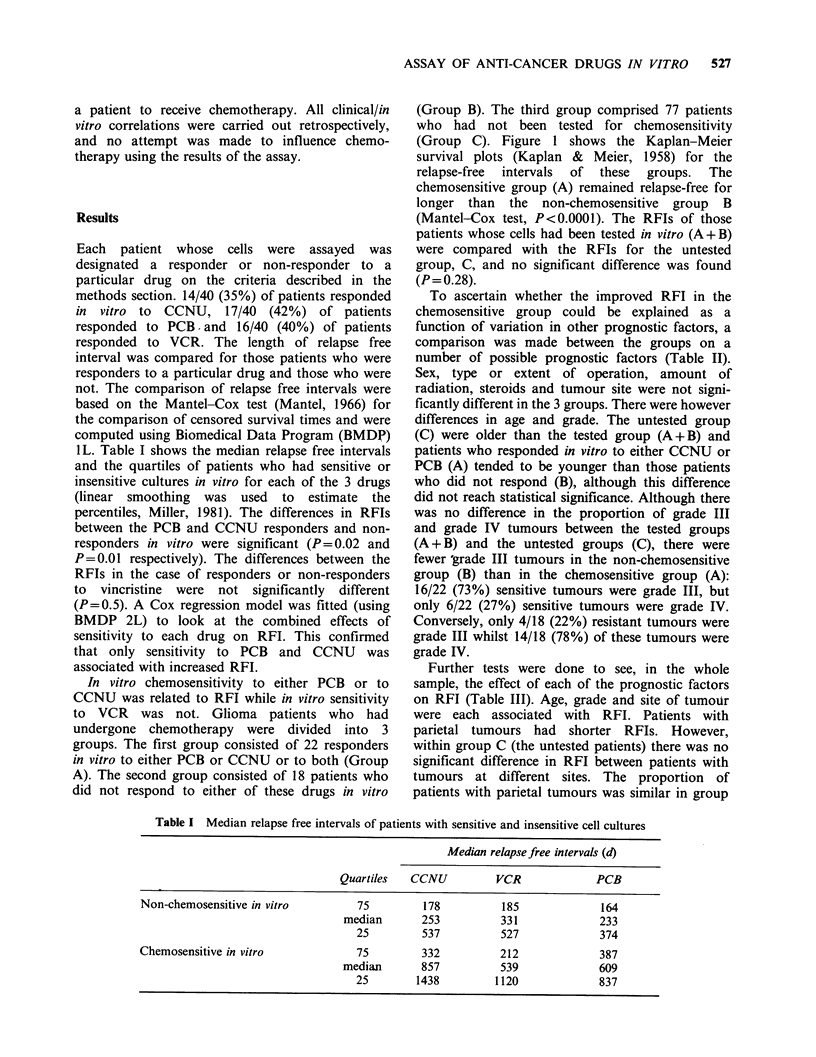

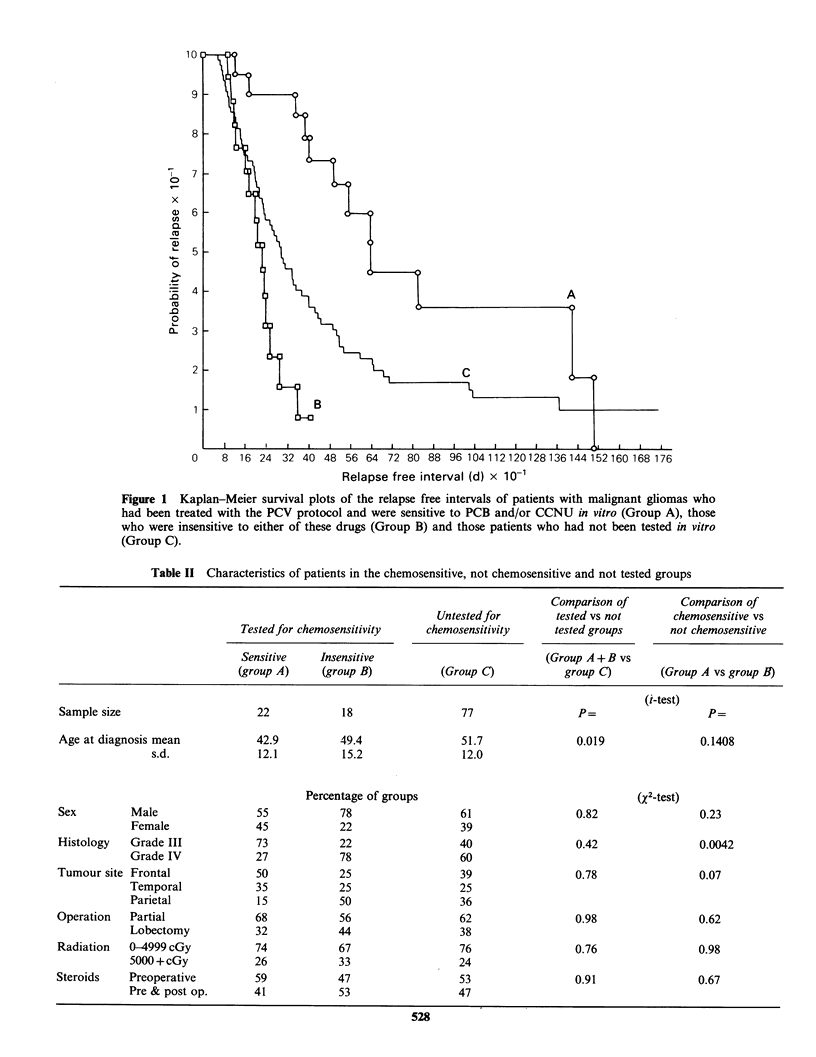

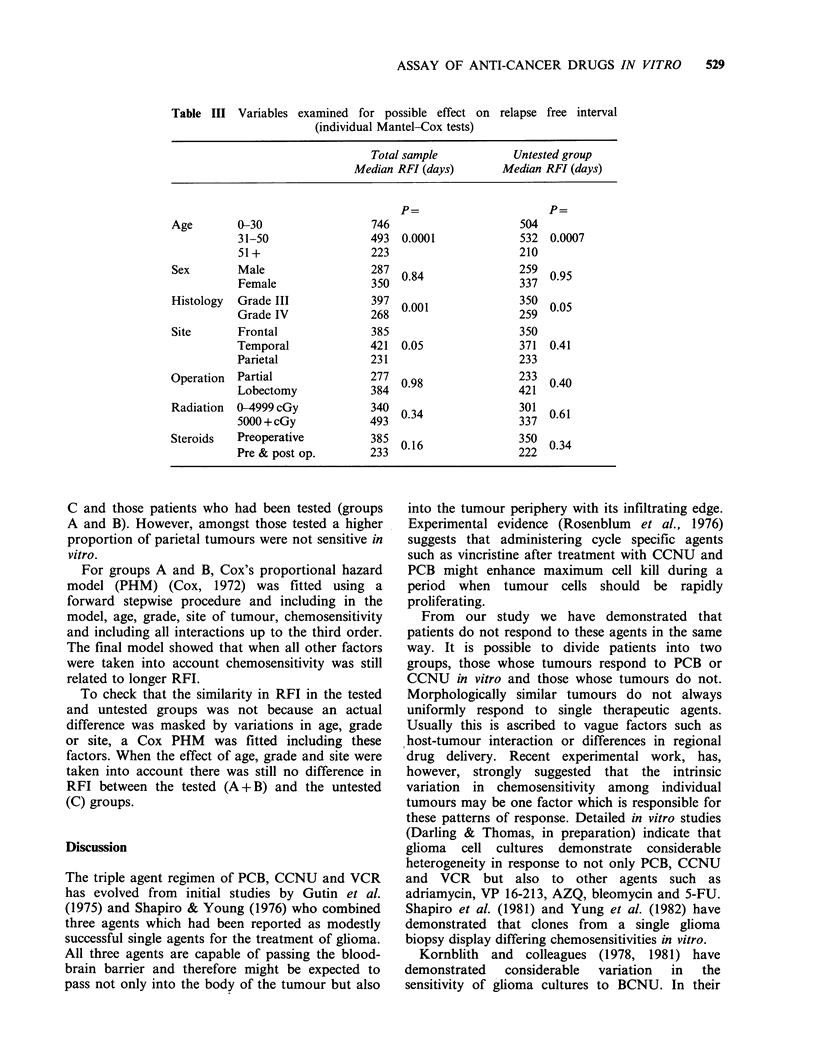

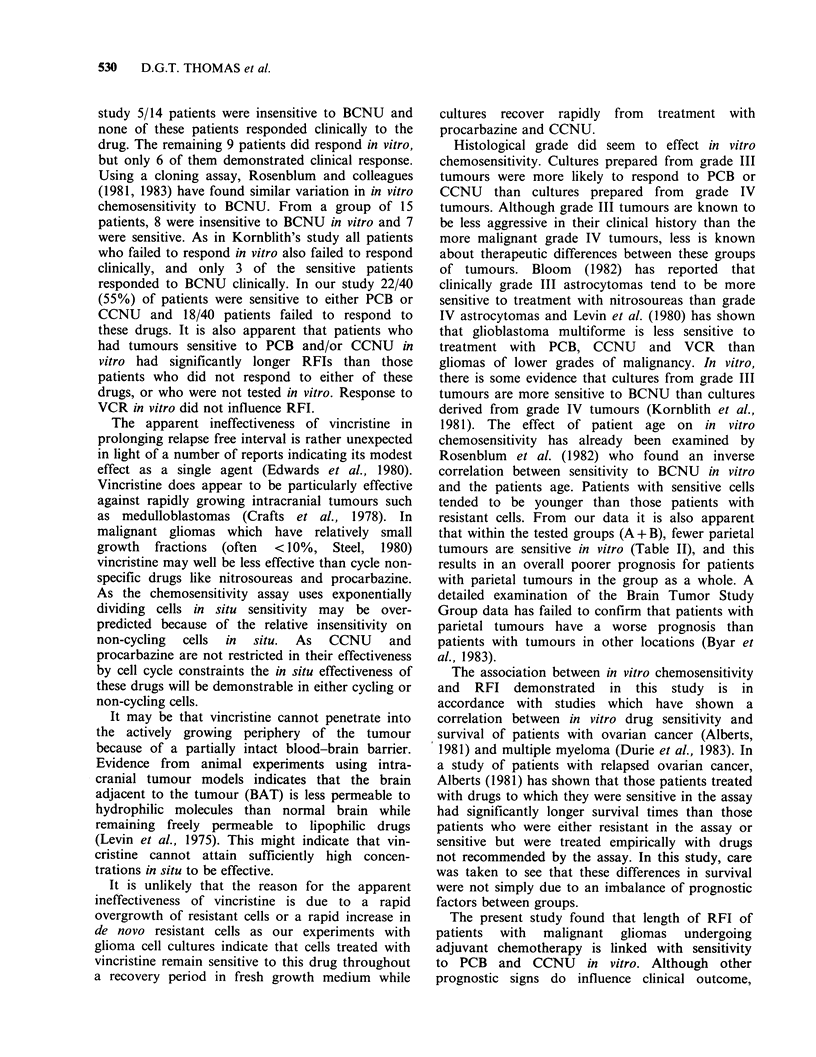

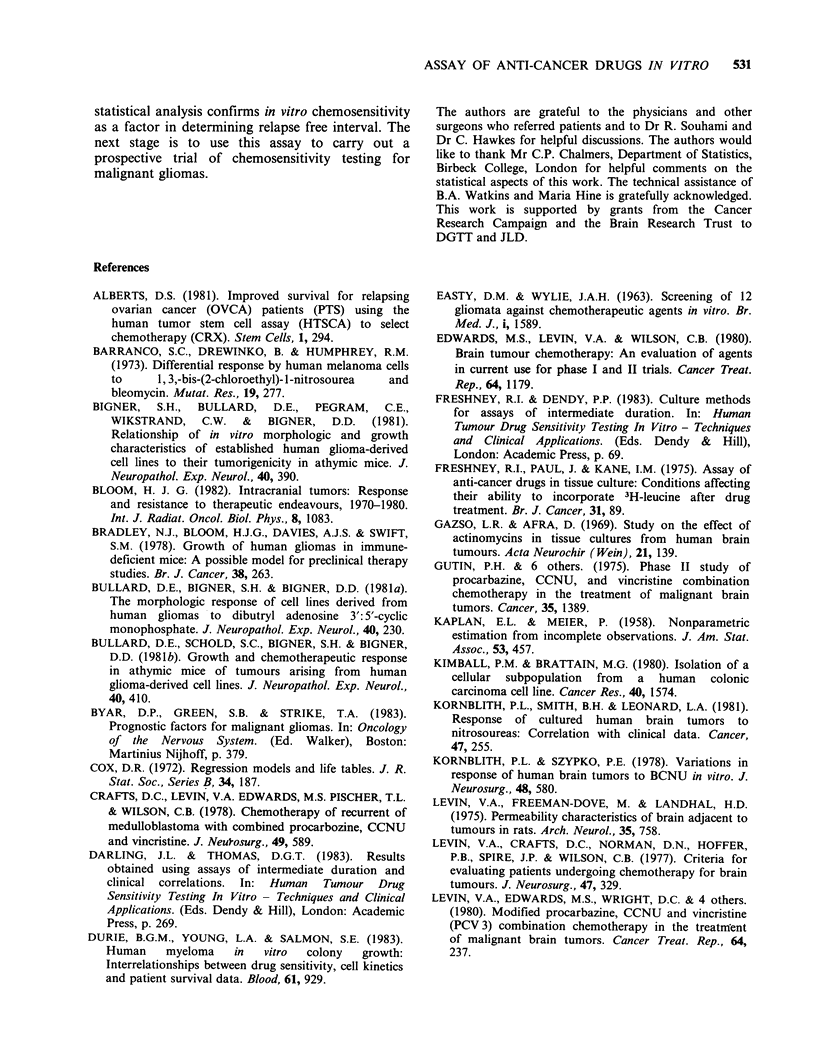

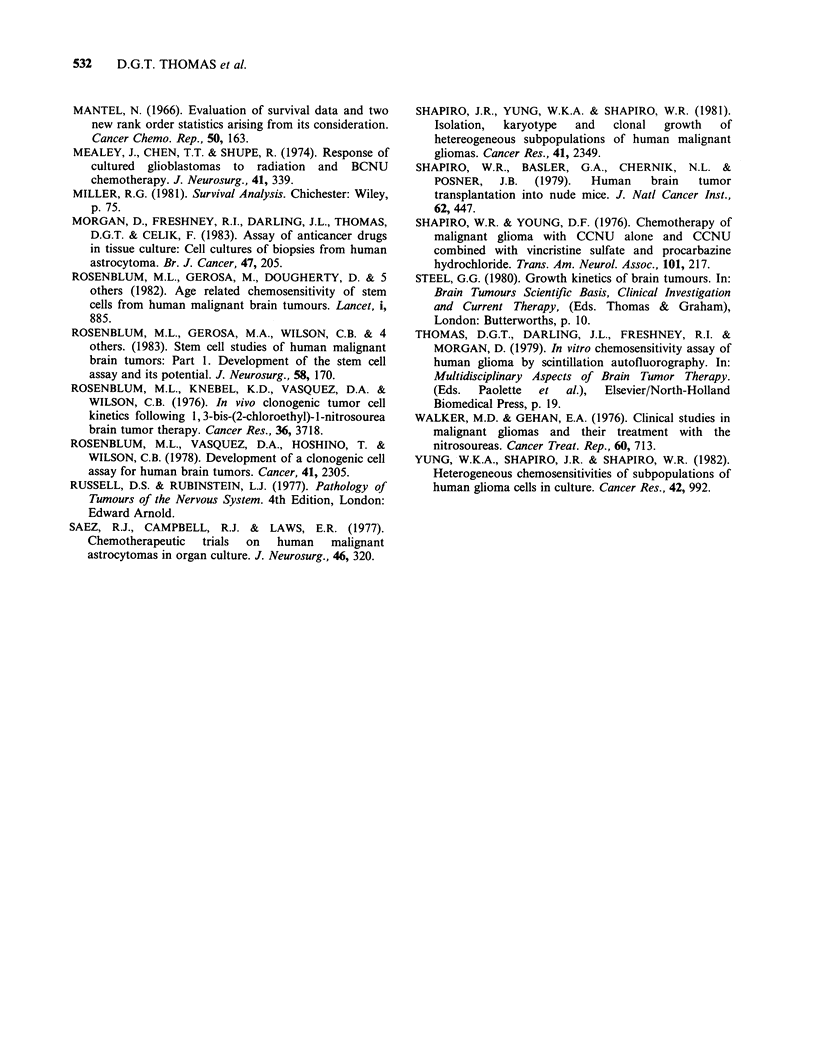


## References

[OCR_00728] Berranco S. C., Drewinko B., Humphrey R. M. (1973). Differential response by human melanoma cells to 1,3-bis-(2-chloroethyl)-1-nitrosourea and bleomycin.. Mutat Res.

[OCR_00734] Bigner S. H., Bullard D. E., Pegram C. N., Wikstrand C. J., Bigner D. D. (1981). Relationship of in vitro morphologic and growth characteristics of established human glioma-derived cell lines to their tumorigenicity in athymic nude mice.. J Neuropathol Exp Neurol.

[OCR_00742] Bloom H. J. (1982). Intracranial tumors: response and resistance to therapeutic endeavors, 1970-1980.. Int J Radiat Oncol Biol Phys.

[OCR_00747] Bradley N. J., Bloom H. J., Davies A. J., Swift S. M. (1978). Growth of human gliomas in immune-deficient mice: a possible model for pre-clinical therapy studies.. Br J Cancer.

[OCR_00753] Bullard D. E., Bigner S. H., Bigner D. D. (1981). The morphologic response of cell lines derived from human gliomas to dibutyryl adenosine 3':5'-cyclic monophosphate.. J Neuropathol Exp Neurol.

[OCR_00758] Bullard D. E., Schold S. C., Bigner S. H., Bigner D. D. (1981). Growth and chemotherapeutic response in athymic mice of tumors arising from human glioma-derived cell lines.. J Neuropathol Exp Neurol.

[OCR_00775] Crafts D. C., Levin V. A., Edwards M. S., Pischer T. L., Wilson C. B. (1978). Chemotherapy of recurrent medulloblastoma with combined procarbazine, CCNU, and vincristine.. J Neurosurg.

[OCR_00789] Durie B. G., Young L. A., Salmon S. E. (1983). Human myeloma in vitro colony growth: interrelationships between drug sensitivity, cell kinetics, and patient survival duration.. Blood.

[OCR_00800] Edwards M. S., Levin V. A., Wilson C. B. (1980). Brain tumor chemotherapy: an evaluation of agents in current use for phase II and III trials.. Cancer Treat Rep.

[OCR_00813] Freshney R. I., Paul J., Kane I. M. (1975). Assay of anti-cancer drugs in tissue culture: conditions affecting their ability to incorporate 3H-leucine after drug treatment.. Br J Cancer.

[OCR_00819] Gazsó L. R., Afra D. (1969). Study on the effect of actinomycins in tissue cultures from human brain tumours.. Acta Neurochir (Wien).

[OCR_00835] Kimball P. M., Brattain M. G. (1980). Isolation of a cellular subpopulation from a human colonic carcinoma cell line.. Cancer Res.

[OCR_00840] Kornblith P. L., Smith B. H., Leonard L. A. (1981). Response of cultured human brain tumors to nitrosoureas: correlation with clinical data.. Cancer.

[OCR_00846] Kornblith P. L., Szypko P. E. (1978). Variations in response of human brain tumors to BCNU in vitro.. J Neurosurg.

[OCR_00856] Levin V. A., Crafts D. C., Norman D. M., Hoffer P. B., Spire J. P., Wilson C. B. (1977). Criteria for evaluating patients undergoing chemotherapy for malignant brain tumors.. J Neurosurg.

[OCR_00862] Levin V. A., Edwards M. S., Wright D. C., Seager M. L., Schimberg T. P., Townsend J. J., Wilson C. B. (1980). Modified procarbazine, CCNU, and vincristine (PCV 3) combination chemotherapy in the treatment of malignant brain tumors.. Cancer Treat Rep.

[OCR_00871] Mantel N. (1966). Evaluation of survival data and two new rank order statistics arising in its consideration.. Cancer Chemother Rep.

[OCR_00876] Mealey J., Chen T. T., Shupe R. (1974). Response of cultured human glioblastomas to radiation and BCNU chemotherapy.. J Neurosurg.

[OCR_00885] Morgan D., Freshney R. I., Darling J. L., Thomas D. G., Celik F. (1983). Assay of anticancer drugs in tissue culture: cell cultures of biopsies from human astrocytoma.. Br J Cancer.

[OCR_00897] Rosenblum M. L., Gerosa M. A., Wilson C. B., Barger G. R., Pertuiset B. F., de Tribolet N., Dougherty D. V. (1983). Stem cell studies of human malignant brain tumors. Part 1: Development of the stem cell assay and its potential.. J Neurosurg.

[OCR_00891] Rosenblum M. L., Gerosa M., Dougherty D. V., Reese C., Barger G. R., Davis R. L., Levin V. A., Wilson C. B. (1982). Age-related chemosensitivity of stem cells from human malignant brain tumours.. Lancet.

[OCR_00903] Rosenblum M. L., Knebel K. D., Vasquez D. A., Wilson C. B. (1976). In vivo clonogenic tumor cell kinetics following 1,3-bis(2-chloroethyl)-1-nitrosourea brain tumor therapy.. Cancer Res.

[OCR_00909] Rosenblum M. L., Vasquez D. A., Hoshino T., Wilson C. B. (1978). Development of a clonogenic cell assay for human brain tumors.. Cancer.

[OCR_00919] Saez R. J., Campbell R. J., Laws E. R. (1977). Chemotherapeutic trials on human malignant astrocytomas in organ culture.. J Neurosurg.

[OCR_00924] Shapiro J. R., Yung W. K., Shapiro W. R. (1981). Isolation, karyotype, and clonal growth of heterogeneous subpopulations of human malignant gliomas.. Cancer Res.

[OCR_00930] Shapiro W. R., Basler G. A., Chernik N. L., Posner J. B. (1979). Human brain tumor transplantation into nude mice.. J Natl Cancer Inst.

[OCR_00936] Shapiro W. R., Young D. F. (1976). Chemotherapy of malignant glioma with CCNU alone and CCNU combined with vincristine sulfate and procarbazine hydrochloride.. Trans Am Neurol Assoc.

[OCR_00956] Walker M. D., Gehan E. A. (1976). Clinical studies in malignant gliomas and their treatment with the nitrosoureas.. Cancer Treat Rep.

[OCR_00961] Yung W. K., Shapiro J. R., Shapiro W. R. (1982). Heterogeneous chemosensitivities of subpopulations of human glioma cells in culture.. Cancer Res.

